# Recent advances of *Sargassum pallidum* in chemical and biological aspects

**DOI:** 10.3389/fphar.2025.1492671

**Published:** 2025-03-19

**Authors:** Ziqin Lei, Xiaoyan Qin, Yan Yang, Min Xu, Nan Zeng

**Affiliations:** ^1^ The Third People's Hospital of Chengdu, The Affiliated Hospital of Southwest Jiao Tong University, Chengdu, China; ^2^ State Key Laboratory of Southwestern Chinese Medicine Resources, Chengdu University of Traditional Chinese Medicine, Chengdu, China

**Keywords:** *Sargassum pallidum*, chemical metabolites, biological activities, polysaccharides, antitumor, antioxidant

## Abstract

*Sargassum pallidum* (Turn.) C.Ag. (SP) is a traditional Chinese marine medicinal material known for its extensive pharmacological activities and is primarily found in coastal regions. With a long history of medicinal use in China, it is commonly employed to treat conditions such as goiter, hyperplasia of mammary glands, hypertension, and obesity. Modern research on its phytochemical metabolites has identified polysaccharides, flavonoids, and lipids as the primary metabolites derived from SP, with polysaccharides being the most extensively studied. Modern pharmacological studies have demonstrated that extracts and secondary metabolites obtained from SP exert various biological activities, including antioxidant effects, antitumor properties, hypolipidemic and hypoglycemic actions, antibacterial activity, and immunomodulatory capabilities. This review aims to serve as a theoretical reference for further utilization and development of functional foods derived from marine resources like SP, summarizing relevant literature from both domestic and international sources. Despite a comprehensive overview of chemical metabolites and pharmacological properties, existing limitations suggest the need for more precise technical tools and additional toxicological and clinical studies to ensure quality, safety, and efficacy.

## 1 Introduction

With the continuous decline in terrestrial biological resources, the trend of “seeking medicine from the ocean” is gaining momentum. Natural marine products, with their unique biological resources and diverse structures, have become vital sources for innovative medicine (Wang et al., 2001). Seaweed, a component of traditional Chinese medicine (TCM), is outlined in the 2020 edition of the Chinese Pharmacopoeia. This particular seaweed is derived from the dried bodies of algae belonging to either *Sargassum pallidum* or *Sargassum fusiforme*, belonging to the *Sargassum* family. The former is classified as “macroalgae”, while the latter is known as “microalgae” (National Pharmacopoeia Committee, 2020).

**FIGURE 1:**
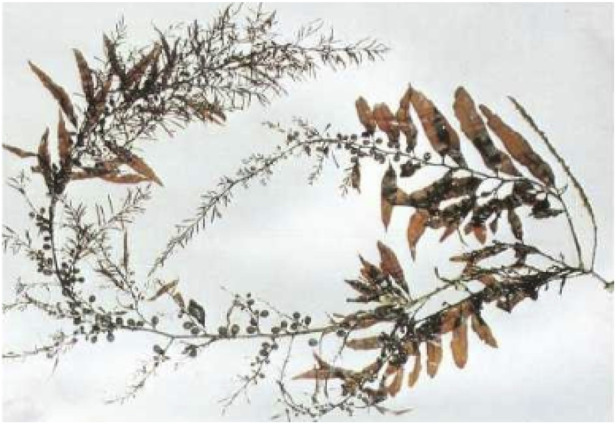
Picture of SP^1^.

*Sargassum pallidum* (Turn.) C.Ag (referred to as SP) is a perennial seaweed belonging to the Sargassoideae subfamily of Rhaeophyta (Li et al., 2021). It is widely distributed in China, Japan, and other Asian countries, primarily found along the coast of the Yellow Sea and Bohai Sea in China (Cao et al., 2014a; Zhang et al., 2022). The plant has a crumpled and curled appearance, growing 30–60 cm in length. Its main stem is cylindrical, with primary branches emerging from both sides and lateral branches arising from the leaf axils of the primary branches. The leaves are simple and alternate; both primary and secondary leaves are lanceolate or linear in shape. The fixation apparatus is discoid, and the black–brown air sacs measure 2–5 mm in diameter and are either spherical or obovate (Qi et al., 2020) (Figure 1) (National Pharmacopoeia Committee, 2020). In China, SP is recognized for its medicinal and culinary properties and is widely consumed. The Dictionary of Traditional Medicine highlights its significant edible and medicinal value (Xiao et al., 2019a). According to the Chinese Pharmacopoeia (National Pharmacopoeia Committee, 2020), SP is characterized by its bitter, cold, and salty properties, affecting the lung, kidney, and spleen meridians. It is believed to have therapeutic effects such as dissolving both soft and hard masses, relieving water retention-related phlegm accumulation, and alleviating symptoms of diarrhea. Historically, physicians have regarded SP as an essential remedy for thyroid adenoma and thyroid gland diseases, which can manifest symptoms such as swelling, accumulation, thyroid adenoma growth, and beriberi testicular swelling edema (Jiangsu New Medical College, 1986). Early coastal farmers discovered that besides preventing goiter development or treating existing goiter (Zeng and Zhang, 1954), SP could also be used as a fertilizer for farmlands. Currently, it is commonly used to treat conditions such as goiter, cervical lymph node tuberculosis, mammary gland hyperplasia, hypertension, and obesity (Zhang et al., 2019a). Chemical studies have identified various compounds in SP, including polysaccharides, flavonoids, and other chemical metabolites (Xu et al., 2011). Furthermore, pharmacological investigations have demonstrated that SP possesses antitumor, antiviral, antioxidant, and antibacterial properties, providing robust scientific support for its traditional medicinal applications (Liu et al., 2012; Zhang Q. et al., 2020).

One of the earliest marine algae used in traditional Chinese medicine, SP is rich in chemical metabolites, drawing increasing interest from researchers studying its pharmacological activities. However, there has been no comprehensive discussion on this topic. Moreover, there is a lack of studies on other metabolites of SP beyond polysaccharides, and the existing studies on its metabolites and pharmacological effects do not align with its broad applications. This highlights the need for greater attention to the nutrients found in SP, including inorganic elements, lipids, polyphenols, and other metabolites, which are expected to impact medicine and health positively.

## 2 Methods

PubMed, Web of Science, Google Scholar, and CNKI were queried for articles on *S. pallidum* published until 30 July 2024. The search terms were related to “*Sargassum pallidum*” and included the following terms: “*Sargassum pallidum,*” “metabolites,” “component,” “pharmacological activity,” “biological activities,” “antioxidant,” “antitumor,” “hypolipidemic,” “hypoglycemic,” “antibacterial,” “antiviral,” and “immunomodulatory.” Conference articles were then excluded. Articles unrelated to *S. pallidum* and studies for which the full text was unavailable were also excluded. Original articles were assessed for scientific validity, and those with scientifically significant findings were retained or otherwise excluded.

ChemDraw 14.0 was used to extract information of the chemical metabolites. The PubChem database (https://pubchem.ncbi.nlm.nih.gov) confirmed the chemical classifications and structures.

## 3 Chemical metabolites of SP

SP encompasses a diverse range of metabolites, primarily including polysaccharides, flavonoids, lipids, nitrogenous compounds, and various inorganic elements (as presented in Table 1). Polysaccharides have emerged as the principal chemical metabolites of SP and have garnered increasing attention in recent years.

**TABLE 1:** Chemical metabolites of *Sargassum pallidum*.

Variety	Number	Metabolites	Rms	Biological activities
Flavonoids	1	2,5-Dihydroxy-6.6′,7,8-tetramethoxyflavone	374.341 g/mol	Anti-tumor (Guo et al., 2009)
	2	5,6-Dihydroxy-7-methoxyflavone	284.263 g/mol	/(Guo et al., 2009)
	3	5,7-Dihydroxy-8-methoxyflavone	284.263 g/mol	/(Guo et al., 2009)
	4	Melanettin	284.263 g/mol	/(Liu et al., 2009)
	5	Stevenin	284.263 g/mol	/(Liu et al., 2009)
	6	Liquirtigenin	256.253 g/mol	/(Liu et al., 2009)
	7	Calycosin	284.263 g/mol	/(Liu et al., 2009; Xu et al., 2013)
	8	Baicalein	270.237 g/mol	/(Xu et al., 2013)
	9	Wogonin	284.263 g/mol	/(Xu et al., 2013)
	10	Quercetin-3-O-glucuronic acid	478.4 g/mol	Antioxidant, hypoglycemic (Xie et al., 2023)
	11	Kuraridin	438.51 g/mol	/(Xie et al., 2023)
	12	Kushenol N	454.51 g/mol	/(Xie et al., 2023)
	13	Formononetin	268.26 g/mol	/(Xie et al., 2023)
	14	Luteolin 7-O-(2-apiosyl-6-malonyl)-glucoside	666.5 g/mol	/(Jiang et al., 2024)
	15	Neohesperidin	610.6 g/mol	/(Jiang et al., 2024)
	16	p-Coumaroyl tartaric acid	296.23 g/mol	/(Jiang et al., 2024)
	17	2′,7-Dihydroxy-4′,5′-dimethoxyisoflavone	314.29 g/mol	/(Jiang et al., 2024)
	18	Daidzin 4′-O-glucuronide	592.5 g/mol	/(Jiang et al., 2024)
	19	Daidzin 7-O-glucuronide	430.4 g/mol	/(Jiang et al., 2024)
	20	Formononetin 7-O-glucuronide	444.4 g/mol	/(Jiang et al., 2024)
	21	Quercetin 3-O-galactoside 7-O-rhamnoside	610.5 g/mol	/(Jiang et al., 2024)
	22	Ellagic acid arabinoside	434.3 g/mol	/(Jiang et al., 2024)
	23	6″-O-Malonyldaidzin	502.4 g/mol	/(Jiang et al., 2024)
Lipids	24	24-Hydroperoxy-24-vinyl-cholesterol	444.690 g/mol	/(Xu et al., 2013)
	25	Fucosterol	412.691 g/mol	/(Xu et al., 2013)
	26	Lecithin	758.060 g/mol	/(Sanina et al., 2004)
	27	L-1-Phosphatidylethanolamine	734.039 g/mol	/(Sanina et al., 2004)
	28	Phosphatidylglycerol	172.074 g/mol	/(Sanina et al., 2004)
	29	Trihydroxyoctadecadienoic acid	328.4 g/mol	/(Xie et al., 2023)
	30	Trihydroxyoctadecenoic acid	330.5 g/mol	/(Xie et al., 2023)
	31	Palmitic acid	256.42 g/mol	/(Xie et al., 2023)
	32	Stearic acid	284.5 g/mol	/(Xie et al., 2023)
Nitrogenous compounds	33	Aurantiamide	402.5 g/mol	/(Guo et al., 2009)
	34	2-amino-3-phenylpropyl acetate	193.242 g/mol	/(Guo et al., 2009)
	35	4-(1H)-quinolinone	145.158 g/mol	Anti-tumour (Guo et al., 2009)
	36	Benzoylphenylalaninol	255.31 g/mol	/(Guo et al., 2009)
	37	2-Benzothiazolol	165.212 g/mol	/(Guo et al., 2009)
	38	(−)-Anabellamide	505.6 g/mol	/(Li C. et al., 2017)
	39	(−)-Trichosanthin	444.522 g/mol	/(Li D. D. et al., 2017)
	40	Riboflavin	376.364 g/mol	/(Li C. et al., 2017)
	41	β-adenosine	267.241 g/mol	/(Li D. D. et al., 2017)
	42	2′-O-Methyluridine	178.231 g/mol	/(Li C. et al., 2017)
	43	Pyrrolo [1,2-a]pyrazine-1,4-dione,hexahydro-3-methyl-, (3 S,8aS)-(9CI)	168.193 g/mol	/(Li D. D. et al., 2017)
	44	Thymidine	242.229 g/mol	Anti-tumor (Li C. et al., 2017)
	45	1-(β-D-ribofuranosyl)-1H-1,2,4-triazone	201.180 g/mol	/(Li D. D. et al., 2017)
	46	Aurantiamide acetate	444.522 g/mol	/(Li C. et al., 2017)
	47	2,3-Dihydro-4(1H)-quinolinone	145.158 g/mol	/(Li C. et al., 2017)
	48	6-Acetyl-7-hydroxy-4-methylquinolin-2-one	217.22 g/mol	/(Xie et al., 2023)
Phenols and phenolic acid	49	6-Gingerol	294.4 g/mol	Antioxidant, hypoglycemic (Xie et al., 2023)
	50	Eckol	372.3 g/mol	Anti-tumor (Xie et al., 2023)
	51	2.2′-Methylenebis (4-methyl-6-tert-butylphenol)	340.5 g/mol	/(Xie et al., 2023)
	52	p-Coumaroyl glycolic acid	222.19 g/mol	/(Jiang et al., 2024)
	53	Carnosol	330.4 g/mol	/(Jiang et al., 2024)
	54	Carnosic acid	332.4 g/mol	/(Jiang et al., 2024)
	55	(−)-Epicatechin	290.27 g/mol	/(Jiang et al., 2024)
	56	(+)-Catechin	290.27 g/mol	/(Jiang et al., 2024)
	57	(+)-Gallocatechin	306.27 g/mol	/(Jiang et al., 2024)
	58	3′-Hydroxy-3,4,5,4′-tetramethoxystilbene	302.32 g/mol	/(Jiang et al., 2024)
	59	Carvacrol	150.22 g/mol	/(Jiang et al., 2024)
	60	Triphlorethol	374.3 g/mol	/(Jiang et al., 2024)
	61	Tetraphlorethol	498.4 g/mol	/(Jiang et al., 2024)
	62	Dihydrocaffeic acid	182.17 g/mol	/(Jiang et al., 2024)
Others	63	2,6,6-trimethyl-4-oxo-2-cyclohexene-1-acetic acid	197.251 g/mol	/(Wang et al., 2010)
	64	2,6,6-trimethyl-4-oxo-2-cyclohexene-1-acetic acid methyl ester	211.277 g/mol	/(Wang et al., 2010)
	65	Monobutyl phthalate	222.237 g/mol	/(Guo et al., 2009)
	66	4-hydroxyphthalide	150.131 g/mol	Anti-tumour (Guo et al., 2009)
	67	Bis(2-methylpropyl)ester	278.34 g/mol	/(Xu et al., 2013)
	68	2-cyclohexene-1-acetic acid	140.180 g/mol	/(Xu et al., 2013)
	69	Dihydroartemisinin ethyl ether I	312.4 g/mol	/(Xie et al., 2023)
	70	1,4-Dimethoxy-2-(2-hydroxydecyl)benzene	294.43 g/mol	/(Xie et al., 2023)
	71	Poricoic acid A	498.7 g/mol	Anti-glycosylation (Xie et al., 2021)
	72	Arctigenin	372.4 g/mol	/(Jiang et al., 2024)
	73	Caffeic acid ethyl ester	208.21 g/mol	/(Jiang et al., 2024)
	74	Gallic acid ethyl ester	198.17 g/mol	/(Jiang et al., 2024)
	75	Pinoresinol	358.4 g/mol	/(Jiang et al. 2024)

### 3.1 Flavonoids

**FIGURE 2:**
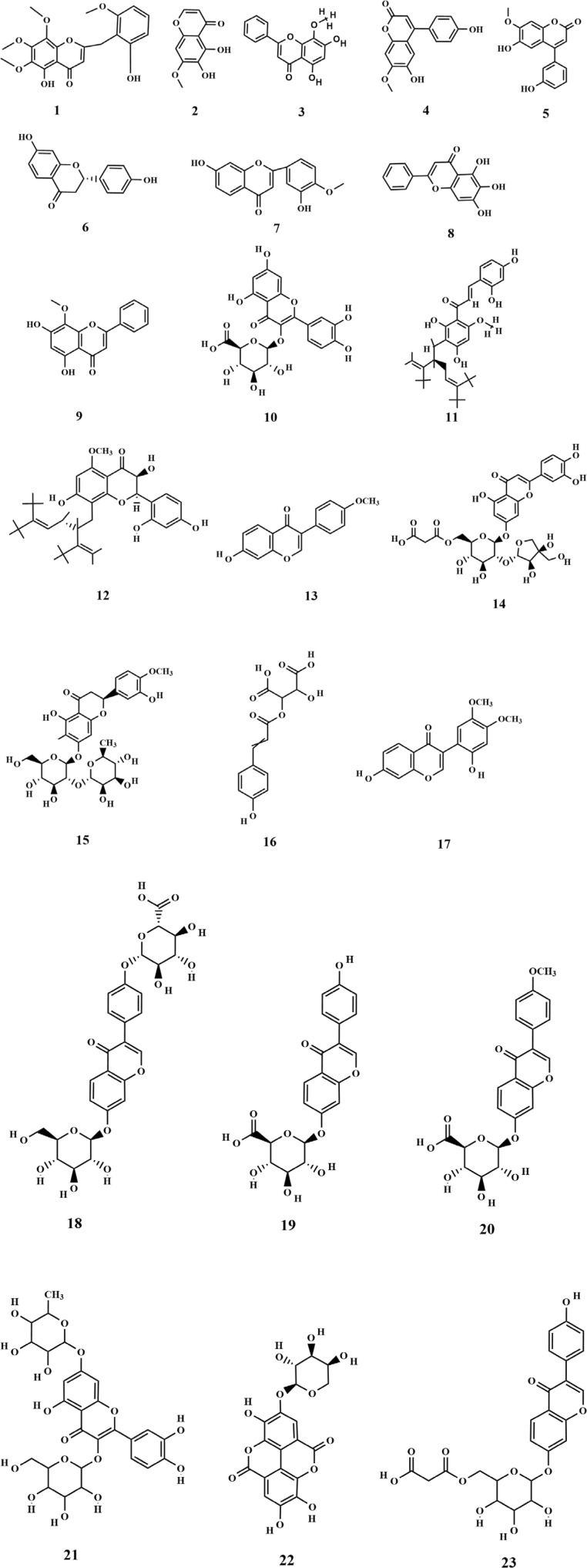
Structure of flavonoids in SP.

Flavonoids are primarily found in terrestrial plants, with relatively few studies focusing on flavonoids derived from seaweed. Three flavonoids (**1-3**) and four isoflavonoids (**4-7**) were isolated for the first time from the ethanolic extract of SP. Among these, 2,5-dihydroxy-6.6′,7,8-tetramethoxyflavone (**1**) was the most abundant one and exhibited significant antitumor activity (Guo et al., 2009; Liu et al., 2009). Furthermore, Xu et al. isolated and characterized three flavonoids from SP, including baicalein (**8**), which was identified as a novel finding with regard to SP and marked a significant advancement in the understanding of SP’s composition (Xu et al., 2013). Subsequent research led to the identification of additional flavonoids (**10-23**), primarily obtained through the preparation of polyphenolic extracts from SP (Xie et al., 2023; Jiang et al., 2024). Among the identified metabolites, quercetin 3-O-glucuronide (**10)** exhibits potent antioxidant and α-glucosidase inhibitory activities, suggesting it may serve as the primary hypoglycemic and antioxidant component in SP polyphenols (Xie et al., 2023). Quercetin 3-O-galactoside 7-O-rhamnoside (**21**) could form hydrogen bonds and interact with the enzyme in question. Moreover, it showed greater binding affinity than the positive drug, illustrating its potential as a potent enzyme inhibitor (Jiang et al., 2024). The chemical structures of these metabolites are depicted in Figure 2.

### 3.2 Polysaccharides

As a crucial class of natural active macromolecules, polysaccharides exhibit diverse activities, including antioxidant, anti-inflammatory, antibacterial, anticancer, antitumor, and immune-enhancing effects. Consequently, they have garnered extensive attention in functional food and biomedicine applications (Xie et al., 2013; Luan et al., 2021). Studies have revealed that SP is abundantly found in active polysaccharides with various beneficial biological effects. The SP polysaccharide structure primarily consists of fucose gum, algal gum, and algal starch (L. B., 2005). Purified SP polysaccharides are predominantly composed of fucose, galactose, xylose glucose mannose, rhamnose, and arabinose (Kong et al., 2022), with glucuronate also present to some extent in the monosaccharide composition of SP (Cao et al., 2019a; Yuan et al., 2020a). Fucose is the most significant monosaccharide component among these metabolites, with the highest molar percentage, 4.01%.

Purified polysaccharides SPI, SPII, and SPIII (SPI: 52.40%; SPII: 51.60%; SPIII: 38.80%, respectively) were isolated and extracted for the first time from SP. Glucose, galactose, mannose, fucose, xylose, fructose, and other monosaccharides were identified as constituents of these polysaccharides (Zhao et al., 2005). Wei et al. successfully isolated and purified three polysaccharide metabolites, P1, P2, and P3, from SP with relative molecular weights of 494,400; 61,500; and 167,600, respectively. The metabolite analysis revealed that the P1 component was primarily composed of xylose and fucose in the following molar percentages: glucose: fucose: galactose: mannose: xylose = 4.3:43.4:12.8:6.2:33.3, respectively; the P2 component mainly consisted of mannose and fucose in the following molar percentages: mannose: fucose: galactose: xylose = 23.8: 44.4: 16.7: 15.1, respectively; the P3 component was predominantly composed of fucose and galactose in the following molar percentages: galactose: fucose: xylose = 27.4:68.9:3.7, respectively (Wei et al., 2007). Ye et al. successfully isolated two polysaccharide metabolites, SP-3-1 and SP-3-2, from SP. The former was found to be composed of glucose, mannose, and galactose in a molar ratio of 11.18:1.00:0.96, respectively, while the latter consisted of fucose, xylose, mannose, glucose, and galactose with a molar ratio of 2.53:0.61:1.00:0.46:0.92, respectively (Ye et al., 2013). The study (Gao et al., 2021) isolated and purified the polysaccharides from SP (SPPs) activities through hot-water extraction, ethanol precipitation, dialysis, and lyophilization. In addition, five polysaccharide fractions (SPP-0.3, SPP-0.5, SPP-0.7, SPP-1, and SPP-25) were obtained, mainly consistent with xylose, galactose, mannose, glucose, rhamnose, and fucose, respectively. After separation, purification, and elution of polysaccharides from SP, a neutral polysaccharide SP-P1 (50.22%) and three acidic polysaccharides, SP-P2 (80.19%), SP-P3 (63.29%), and SP-P4 (77.94%), exhibiting varying degrees of antioxidant activity as well as antitumor and hypoglycemic effects, were obtained, of which monosaccharide components include d-fucose, rhamnose, galactose, glucose, xylose, mannose, and arabinose (Li, 2015; Li C. et al., 2017; Cao et al., 2018). Li et al. used infrared spectroscopy and the PMP pre-column derivatization method to determine the structural metabolites of SP polysaccharides. Their findings revealed that the total sugar content was 24.32%, with a sulfate content of 5.39%. Monosaccharide analysis indicated that the main metabolites from SP polysaccharides samples collected along different regions were galactose, fucose, and mannose (Li et al., 2020). Zhao et al. isolated and purified polysaccharide metabolites DEI and DEII from crude polysaccharides of SP. Their monosaccharide metabolites were glucose, galactose, fucose, fructose, xylose, and mannose (Zhao, 2004), of which fucose had the highest content (DEI: 39.3%; DEII: 35.2%). On the other hand, the hot water extraction method was used to extract crude polysaccharides from SP, followed by separation and purification, resulting in sulfated fucosan SF0. This metabolite primarily comprised mannose, glucuronate, glucose, galactose, xylose, and fucose in a molar ratio of 10.7:8.3:3.0:21.1:21.2:35.6, respectively (Xue et al., 2023). The monosaccharide metabolites of SPPs also vary depending on their geographical origins. Polysaccharides isolated from SP collected along the Weihai, Shandong Province coast consist of galactose, xylose, fucose, mannose, and glucuronate. On the other hand, polysaccharides derived from Laminaria japonica in Yantai are composed of xylose, fucose, galactose, fructose, mannose, and glucose (Cao et al., 2014b).

### 3.3 Lipids

**FIGURE 3:**
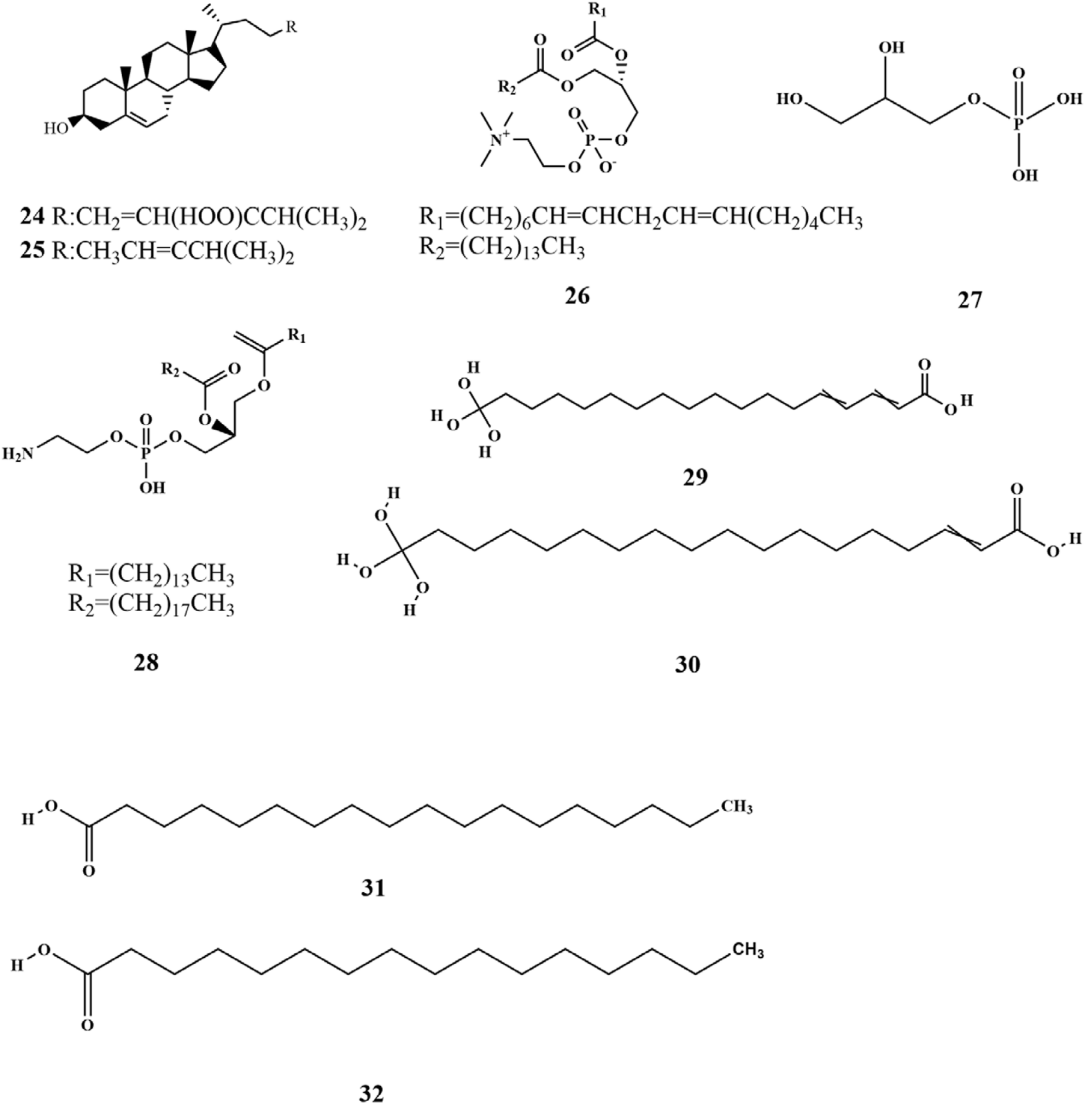
Structure of lipids in SP.

Research has shown that a limited quantity of sterol metabolites is present in SP. Pharmacological investigations demonstrated that phytosterols possess preventive effects against cardiovascular diseases and have antitumor properties, which highlights the significance of sterol metabolites found in SP (Xu et al., 2013). Xu et al. isolated two sterol compounds from SP (**24–25**), of which fucosterol (**25**) plays a crucial role in maintaining internal homeostasis in organisms. It helps in regulating glycogen and mineral metabolism, preventing and treating cancer, and reducing blood cholesterol levels (Xu et al., 2013). Phospholipid metabolites (**26–28**) have been isolated from SP (Sanina et al., 2004). SP is also rich in fatty acids, closely associated with human health. Fatty acids are carboxylic acids with the general structure of RCOOH, containing a methyl end, a hydrocarbon chain (R), and a carboxylic terminus. Wang et al. identified various fatty acid metabolites from the fat-soluble constituents of SP using gas chromatography, including C12:0, C14: 0, C16: 0, C16: 1W7, C18: 0, C18: 1W9, C18: 2W6, C18: 3W3, C20: 1W7, C20: 3W6, C20: 4W6, C20: 5W3, and C22: 1W11 (Wang et al., 2001). The fatty acids were categorized into saturated and unsaturated acids, with their total mass fraction accounting for 3.45% of dried algae. However, the content of unsaturated fatty acids was significantly higher than that of saturated ones (Li et al., 1998; Wang et al., 2001). Four kinds of fatty acids (**29–32**) were obtained from soluble polyphenols of SP (Xie et al., 2023). The chemical structures are depicted in Figure 3.

### 3.4 Nitrogenous metabolites

Alkaloids are nitrogen-containing alkaline organic compounds known for their specific biological activities, making them a central focus of research on natural products. Guo et al. isolated and identified five structural types of alkaloids (**33–37**) from the ethanolic extract of SP. Among them, aurantiamide (**33**) was the most abundant and demonstrated no significant biological activity, while 4-(1H)-quinolonone (**35**) exhibited notable inhibitory activity against the K562 cell cycle (G0/G1 phase) (Guo et al., 2009). Subsequently, Li et al. isolated ten additional nitrogenous metabolites (**38–47**) from the ethanolic extract of SP, among which aurantiamide acetate (**46**) was the most abundant, accounting for 20.8%. Eight metabolites (**38–45**), including (−)-anabellamide, were isolated for the first time from the *Sargassum* family (Li D. D. et al., 2017).

**FIGURE 4:**
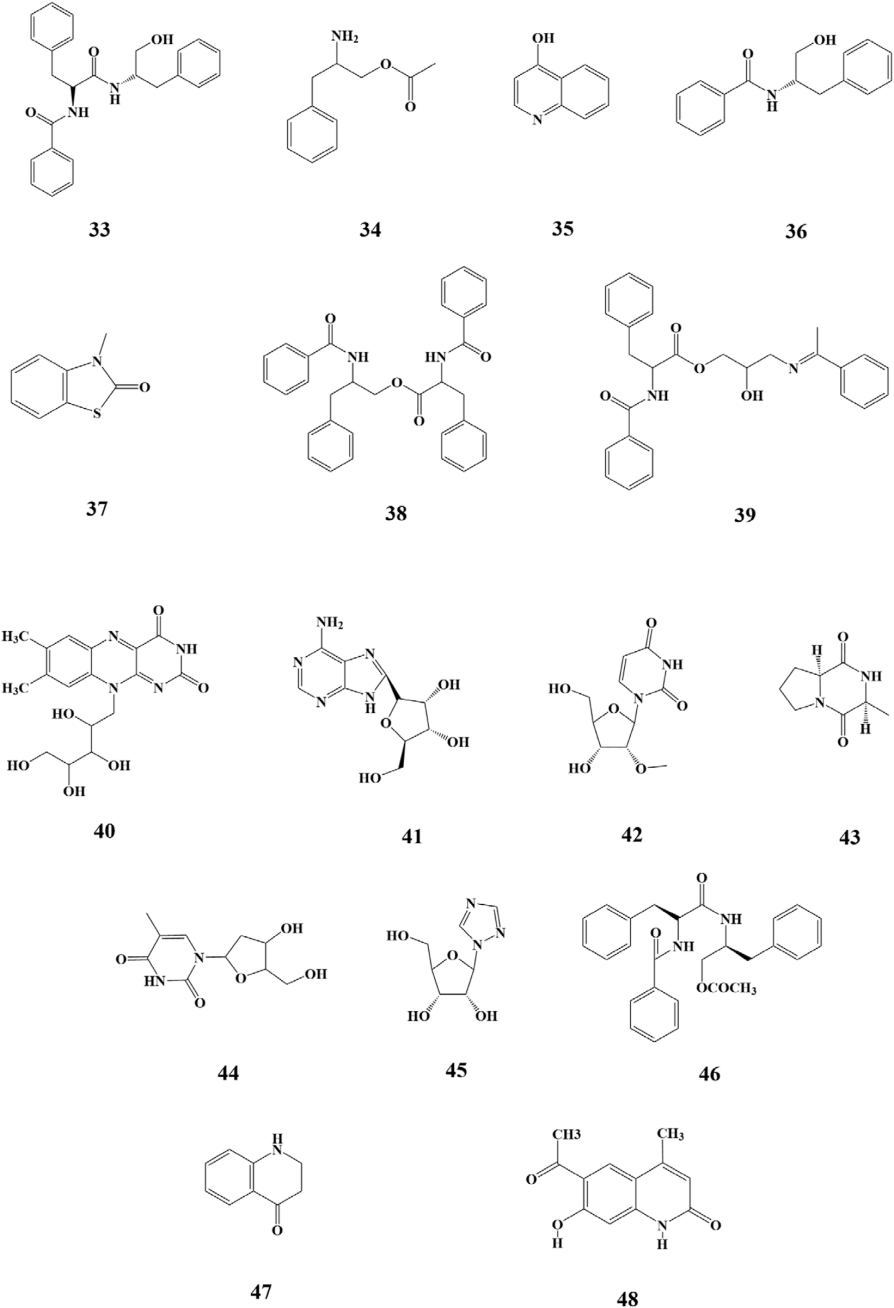
Structure of nitrogenous metabolites in SP.

Additionally, 6-acetyl-7-hydroxy-4-methylquinolin-2-one (**48**) was identified in the polyphenol extract of SP (Xie et al., 2023). The chemical structures are depicted in Figure 4.

### 3.5 Phenols and phenolic acid

**FIGURE 5:**
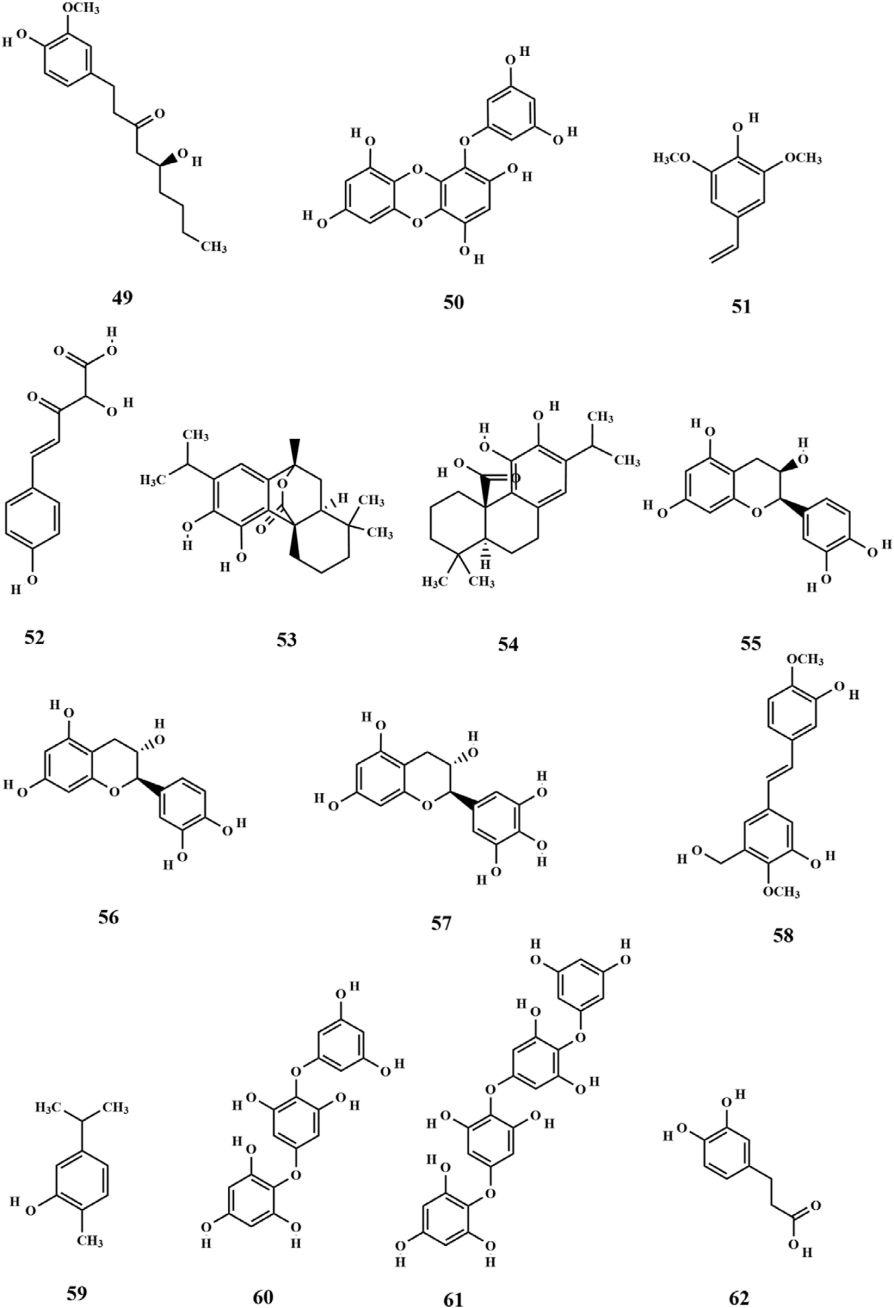
Structure of phenols and phenolic acids in SP.

The polyphenol extract of SP was prepared using SP as the raw material. The chemical composition of the polyphenol fractions of SP was identified (**49–62**), which includes 6-gingerol (Xie et al., 2023; Jiang et al., 2024). 6-Gingerol (**49**) exhibits potent antioxidant and hypoglycemic activities, demonstrating high response values in mass spectrometry of polyphenol fractions. This may contribute to the extracellular activity of SP (Xie et al., 2023). In addition, the polyphenols of SP have suitable antimicrobial and antioxidant activities, possibly owing to their phenolic structure. The chemical structures are depicted in Figure 5.

### 3.6 Others

**FIGURE 6:**
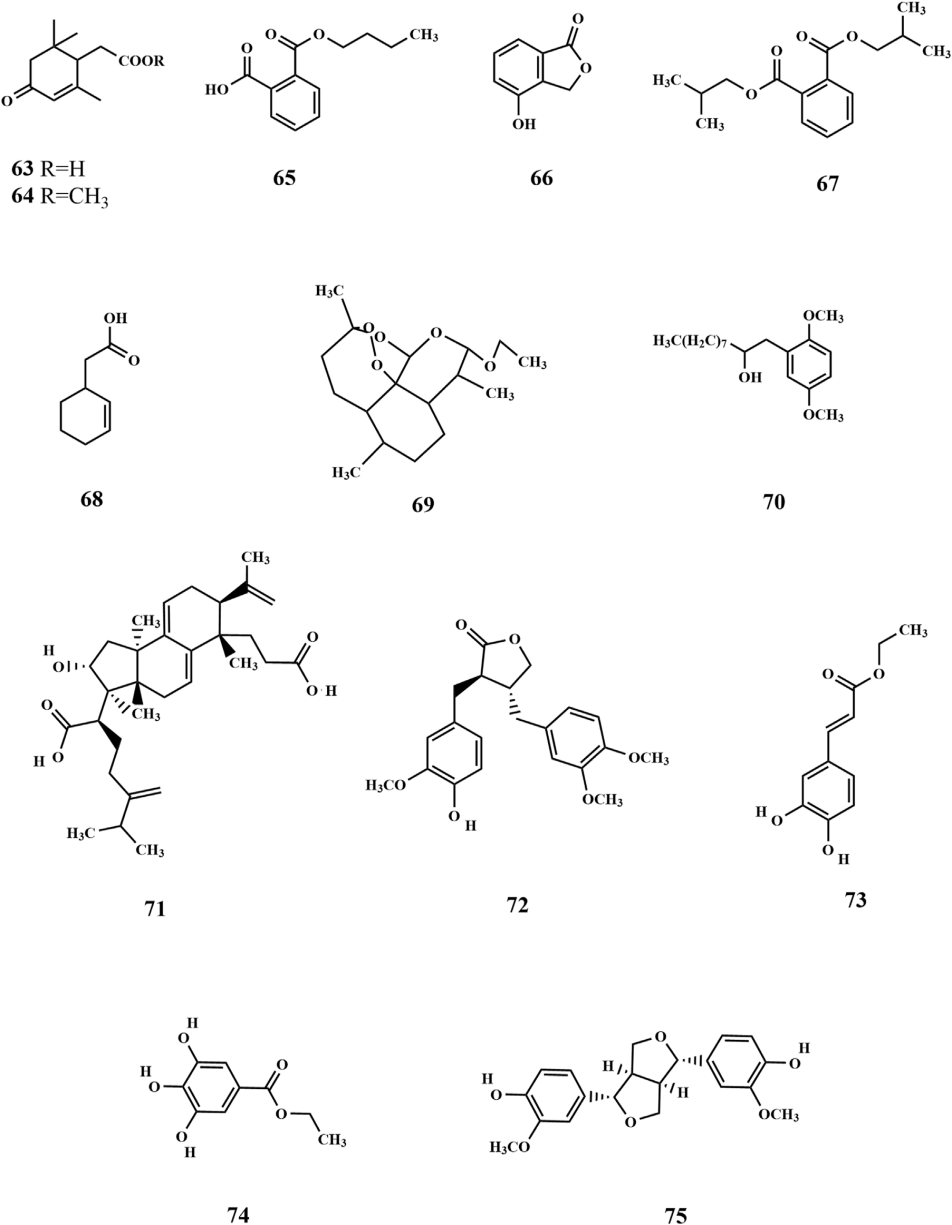
Structure of others in SP.

Metabolic organic acids act as intermediates in the metabolism of biological substances, with ketone acids playing a crucial role in energy and amino acid metabolism. The body can produce ketoacids *via* the glycolytic pathway or the liver, which are transported for energy using the bloodstream to other tissues. When the body’s energy requirements are high, muscles and other tissues can oxidize ketoacids to produce ATP to meet the energy needs. Currently, ketone acid compounds such as 2,6,6-trimethyl-4-oxo-2-cyclohexene-1-acetate (**63**) and 2,6,6-trimethyl-4-oxo-2-cyclohexene-1-acetate methyl ester (**64**) have been isolated from SP (Wang et al., 2010). Guo et al. were the first to isolate monobutyl phthalate (**65**) and 4-hydroxyphthalide (**66**) from the ethanolic extract of SP. 4-Hydroxyphthalide is an antitumor active ingredient of SP with a significant inhibitory effect on the P388 cell cycle (G0/G1 phase) (Guo et al., 2009). The following compounds were identified: bis(2-methylpropyl) ester (**67**), 2-cyclohexene-1-acetic acid (**68**) (Xu et al., 2013), dihydroartemisinin ethyl ether I (**69**), 1,4-dimethoxy-2-(2-hydroxydecyl) benzene (**70**) (Xie et al., 2023), poricoic acid A (**71**) (Xie et al., 2013), arctigenin (**72**), caffeic acid ethyl ester (**73**), gallic acid ethyl ester (**74**), and pinoresinol (**75**) (Jiang et al., 2024). Of these, poricoic acid A (**71**) has been identified as a promising candidate for antiglycation inhibition, with potential applications in managing diabetes-related complications by incorporation of this compound into dietary products. Apart from these components (Figure 6) mentioned above, there are a few research reports on its active proteins, with one specific protein having a molecular weight of 46 kD that has been successfully isolated (Xu et al., 2011). It still contains inorganic elements K, Mg, Ca, Na, Cd, As, Al, Cu, Hg, and Pb, and the content of constant elements K, Mg, Ca, and Na is high (Cao et al., 2014a).

## 4 Biological activities

**FIGURE 7:**
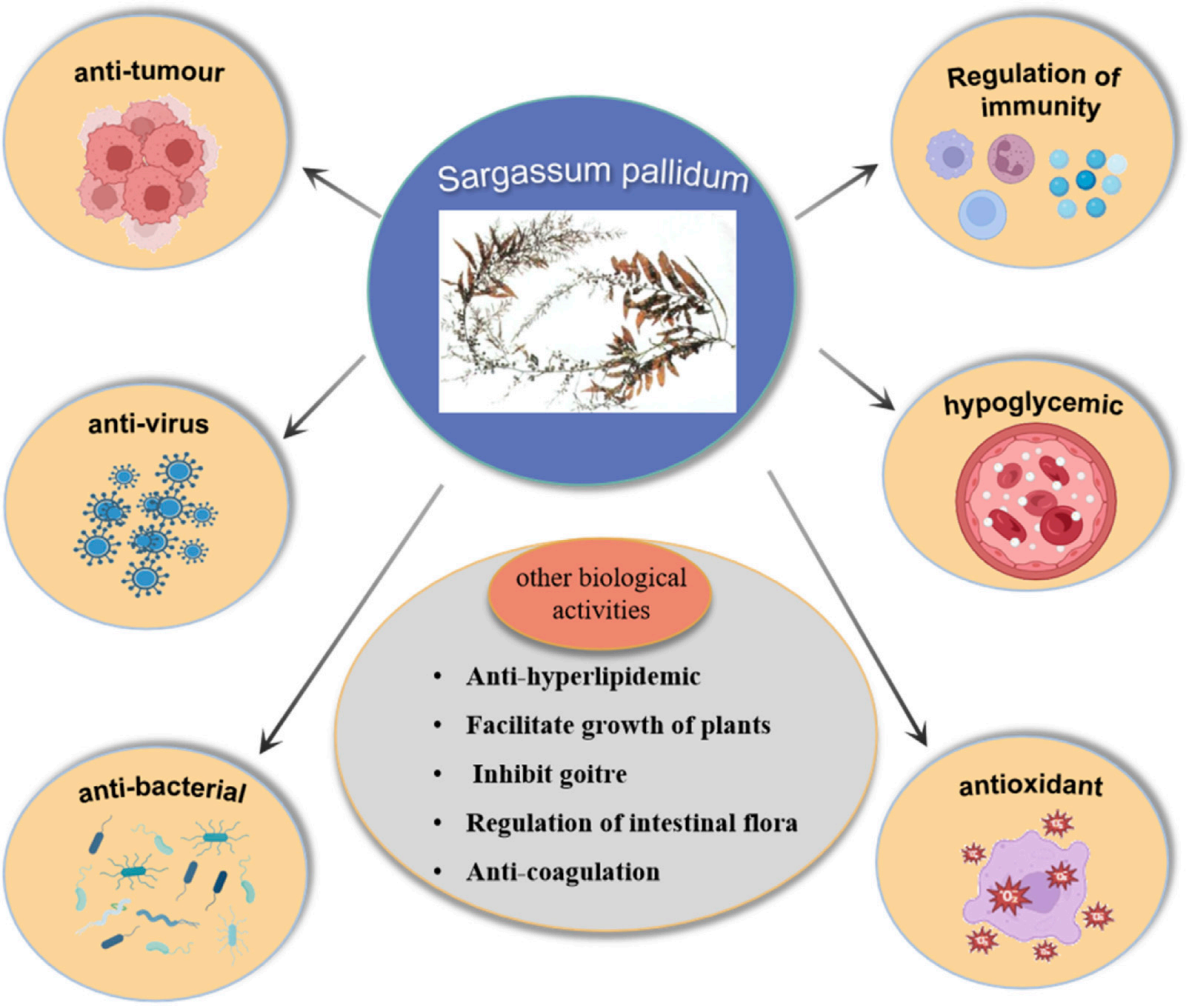
Biological activities of SP.

Modern studies have shown that the metabolites isolated from SP exhibit a variety of biological activities, including antitumor, antiviral, antibacterial, hypoglycemic, antioxidant, and immunomodulatory activities, as illustrated in Figure 7.

### 4.1 Antitumor effects

*In vivo* and vitro experiments have shown that the chemical metabolites of SP can exert antitumor effects by inhibiting cancer cell proliferation and promoting tumor cell apoptosis. Guo et al. evaluated the antitumor activity of 11 isolated metabolites using flow cytometry and cell proliferation inhibition assays *in vitro*. The results showed that the SP chemical fraction thymidine exhibited potent necrotoxic and cytotoxic activity against tsFT210 cells (Guo et al., 2009). Specifically, the metabolites 2′,5-dihydroxy-6,6′,7,8-tetramethoxyflavone (0.13 μmol/L) and 4-quinol-one (0.34 μmol/L) demonstrated significant cell cycle inhibitory activity against K562 cells. Furthermore, both 2′,5-dihydroxy-6,6′,7,8-tetramethoxyflavone and 4-hydroxyphthalide significantly inhibited cell cycle activity in P388 cells at concentrations of 0.13 and 0.33 μmol/L, respectively.

In addition, SP polysaccharide (SPP) at a concentration of 500 μg/mL showed enhanced anti-proliferative effects on HepG2 tumor cells. The inhibition of human cervical squamous cell carcinoma SiHa cell proliferation was observed at a concentration ranging from 0.125 to 0.5 mg/mL and was concentration-dependent, with the strongest inhibitory effect observed at the dosage of 0.5 mg/mL (Li., 2015). The SPP fractions SP-1, SP-2, and SP-3 exerted antitumor activity in a dose-dependent manner (0.125–1.00 mg/mL), inhibiting the growth rates of human hepatocellular carcinoma cell line HepG2, human lung carcinoma cell line A549, and human gastric carcinoma cell line MGC-803. The highest inhibition rate of SP-3 on the growth of A549, HepG2, and MGC-803 cells was observed at a concentration of 1.00 mg/mL (Ye et al., 2008). Liu et al. intraperitoneally injected S180 sarcoma mice with SPPs (50, 100, and 200 mg/kg) for 10 consecutive days. They found that the tumor growth rate was inhibited, and the mRNA and protein expression levels of oncogenes p53 and Rb were significantly upregulated, suggesting that SPP could hinder the growth of S180 sarcoma *in vivo* (Liu and Meng, 2008). Li et al. treated A549 tumor cells with SPPs (0–400 μg/mL) and discovered an inhibitory effect on tumor cell proliferation, with the most substantial impact observed at 100 μg/mL. Furthermore, SPP enhanced the Bax/Bcl-2 ratio, thereby facilitating apoptosis in tumor cells and contributing to its antitumor effect (Li et al., 2020). Gao et al. discovered that all five SPP fractions had significant antitumor activity against cancer cells A549, HepG2, and B16 and could induce apoptosis in cancer cells. Transcriptome sequencing results showed that SPP-0.7 (25 μg/mL), the most active fraction, significantly induced cancer cell apoptosis, cytokine secretion, and cellular stress response process while inhibiting the normal physiological processes of cancer cells. Overall, SPP and SPP-0.7 may serve as promising candidates for cancer therapy (Gao et al., 2021). Generally speaking, SP plays a positive role in *in vitro* and *in vivo* models of various cancers such as lung, liver, gastric, and cervical cancer. We summarized the main mechanisms by which SP exerts anticancer activity: 1. directly affecting the viability of tumor cells or exerting cytotoxic effects and 2. inhibiting the progression of the cell cycle of tumor cells by regulating cell cycle-related proteins and genes to affect their division and proliferation. Follow-up studies could focus on whether the antitumor effect could be exerted by regulating other cellular functions or the secretion of certain adhesion factors and also concentrate on specific classical apoptotic pathways, such as caspase-mediated signaling pathways, for further mechanistic studies.

### 4.2 Antiviral effects

Heparin is a glycosaminoglycan consisting of variably sulfated repeating disaccharide units, which can be extracted from seaweed. Several strains of human papillomavirus (HPV) have been reported to bind heparan sulfate as a low-affinity co-receptor. High-risk HPV16 can attach to heparan sulfate on the cell surface *via* the viral envelope protein L1, and it cannot infect cervical cells without this molecule. Heparan sulfate can also mimic the receptor for HPV (Xiao et al., 2022). The SPP metabolite fucoidan is a marine heparin-like polysaccharide that can effectively inhibit HPV infection *in vitro*, with an IC50 value of 1.1 μg/mL (Moga et al., 2021). The antiviral activity of fucoidan from diverse sources has been demonstrated *in vivo* and *in vitro*. Fucoidan has the ability to hinder the adsorption of viruses, thereby preventing the formation of syncytia. It can also directly inhibit viral replication and stimulate both innate and adaptive immune responses. The antiviral activity of fucoidan against the influenza A virus (H1N1) has been evaluated using a cytopathic effect assay. The sulfated fucoidan 3(SF3) exhibited the most excellent inhibitory effect at 250 μg/mL. Although less active than the positive control drug ribavirin, this fucoidan still has potential antiviral value due to its low cytotoxicity (Xue et al., 2023). In summary, SPPs can interfere with virus adsorption and penetration. However, their intrinsic mechanism of action remains unclear, which presents a promising area for future research.

### 4.3 Antibacterial effects

Studies on the antimicrobial activity of SP have primarily focused on SP extracts or polyphenolic components. Lin et al. found that both ethanol and ether extracts of SP exhibited significant antibacterial effects against *Bacillus subtilis*, *Escherichia coli*, and *Staphylococcus aureus*. Additionally, the ethyl acetate extract showed inhibitory effects against *Penicillium* species (Lin et al., 2011). In a follow-up study (Lin et al., 2012), the extracted crude lipids from SP were further fractionated, and the antibacterial activity of each fraction was determined. The inhibition diameter of SP crude lipids against *Pseudomonas aeruginosa* and *E. coli* were measured at 1.5 mm and 1.0 mm, respectively, while showing no effects on *S. aureus* and *B. subtilis*. The inhibition circles of the ethanol-eluting fraction against *P. aeruginosa*, *E. coli*, *S. aureus*, and *B. subtilis* were recorded at 2.5, 4.5, 4.0, and 3.0 mm, respectively. Meanwhile, the benzene-eluted fraction had an inhibition diameter of 1.0 mm against *B. subtilis* and *S. aureus* but showed no inhibitory activity against *E. coli* and *P. aeruginosa*. The petroleum ether-eluted fraction had no inhibitory activity against these four bacteria, suggesting that the ethanol-eluted fraction of SP possesses more potent antibacterial properties.

Seaweed polyphenols, mainly phenolic derivatives, have a variety of biological activities. Dang et al. found that SP polyphenols displayed significant inhibitory effects on *Vibrio* neptunculus, *Vibrio* brasiliensis, *Vibrio* Xu, *Vibrio* Chagas, *Vibrio* Eel, *Vibrio Bordetella*, and *Vibrio* harveyi. It was observed that the greater the polyphenol content compared to that found in other algae, such as *Hordeum vulgare* and *Sargassum*, the stronger the inhibitory effect (Dang et al., 2012). As such, the presence of phenolic hydroxyl groups may be the key to the antimicrobial effects of SP, and greater attention should be paid to its conformational relationship in future research.

### 4.4 Hypolipidemic and hypoglycemic effects

Zhang et al. showed that SPP could significantly reduce the serum levels of total cholesterol and triglycerides in hyperlipidemic mice at a dose of 4.0 g/kg. As the dose was increased, the lipid-lowering effect in hyperlipidemic mice was enhanced. Additionally, SPP improved the increased liver index and decreased renal coefficient in mice caused by a high-fat emulsion. This may be related to the presence of a large number of active groups, such as sulfate, in SPPs (Zhang et al., 2009). In addition, some scholars found that an ethanol extract of SP (3%) was effective in reducing adipose tissue weight in obese rats. This extract significantly lowered triglyceride, total cholesterol, and fatty acid contents of the model rats, suggesting that the ethanol extract of SP has the potential to lower blood lipids and help control obesity (Yu-Jung et al., 2011). Lin et al. found SP fucoidan evidently decreased body weight, hyperlipidemia, and hyperglycemia in mice while increasing insulin sensitivity. They found that SP fucoidan could ameliorate the glucose and lipid metabolism disorders in diabetic mice by activating Nrf2/ARE antioxidant signaling in the diabetic complication study (Lin et al., 2023).

The human body contains numerous carbohydrate-hydrolyzing enzymes, with α-amylase and α-glucosidase being the most important (Wu et al., 2016). Inhibiting their activities can significantly slow the conversion of glucose to blood glucose, leading to lower postprandial blood glucose levels, making it an effective treatment for type 2 diabetes (Heacock et al., 2005; Kim, 2013; Rodrigues et al., 2017). Cao et al. explored the hypoglycemic activity of polysaccharide *in vitro* experiments and found that the SPP-2 (5.0 mg/mL) and SPP-1 (2.0 mg/mL) effectively inhibited α-amylase and α-glucosidase, respectively (Cao, 2021). Yang et al. evaluated the antidiabetic effects of alginate oligosaccharides in a hamster model fed a high-fat and high-sucrose diet. It was found that administration of alginate oligosaccharides significantly reduced fasting blood glucose levels. This therapeutic effect was attributed to the modulation of the insulin receptor substrate 1/phosphatidylinositol 3-kinase (IRS1/PI3K) and c-Jun N-terminal kinase (JNK) pathways. Meanwhile, high-throughput 16S rRNA gene sequencing analyzed the gut flora composition in diabetic animals, revealing that alginate oligosaccharides notably increased the relative abundance of *Lactobacillus* and *Clostridium* XIVa, while decreasing that of the homologous bacilli *Bacteroides anthropophilus* and *Clostridium* IV. It suggests that algal oligosaccharides may alleviate obesity and exhibit antidiabetic properties by partially regulating the gut flora in diabetic patients (Yang et al., 2019). Xie et al. used high-fat diet and streptozocin (HFD/STZ)-induced T2DM mice as a model to explore the modulation effects of SP extract on hyperglycemia and hyperlipidemia. Results indicated that SPPE could significantly regulate dyslipidemia. The IRS1/PI3K/Akt pathway is critical for maintaining glucose homeostasis (Deng et al., 2020). Xie et al. revealed that SPPE affects this pathway by modulating the expression of essential genes related to the PI3K/Akt pathway and lipogenesis in the liver (Xie et al., 2023). Numerous studies have shown sulfated derivatives possess superior hypoglycemic properties compared to natural SPPs. Experimental results showed that SPP and sulfated S-SPP1-4, S-SPP1-6, and S-SPP1-8 exhibited a dose-dependent inhibition of α-glucosidase within the concentration range of 0.0625–1.0 mg/mL. Among them, sulfated SPP showed better inhibition of α-glucosidase activity and promotion of glucose consumption in insulin-resistant cells, suggesting that sulfated SPP could serve as an alternative hypoglycemic agent (Xiao et al., 2019b). SPP-1 and SPP-2 dose-dependently inhibited α-amylase and α-glucosidase activities in the 0.125–5.0 mg/mL concentration range, with SPP-1 showing greater inhibition of α-amylase than acarbose, highlighting its potential as a promising α-glucosidase inhibitor (Cao et al., 2018). Selenopolysaccharides combine the benefits of selenium and polysaccharides, and modification of polysaccharides with inorganic selenium can improve the effective delivery of selenium. Selenium modification of SPP was found to have a more pronounced inhibitory effect on α-glucosidase (Xiao et al., 2019a). It can provide a reference basis for the development of functional foods. Polyphenols are small-molecule compounds containing one or more phenolic hydroxyl groups and are widely distributed in fruits, vegetables, nuts, seeds, flowers, and barks (Cutrim and Cortez, 2018). It has been reported that polyphenols such as flavonoids, which are a good source of hypoglycemic agents with high efficiency, low toxicity, and minor side effects, can delay the absorption of glucose by inhibiting the activity of α-glucosidase (Papoutsis et al., 2021). A recent study showed that SP polyphenols inhibited α-glucosidase activity, reflecting its good hypoglycemic activity (Xie et al., 2023; Xie et al., 2024).

**FIGURE 8:**
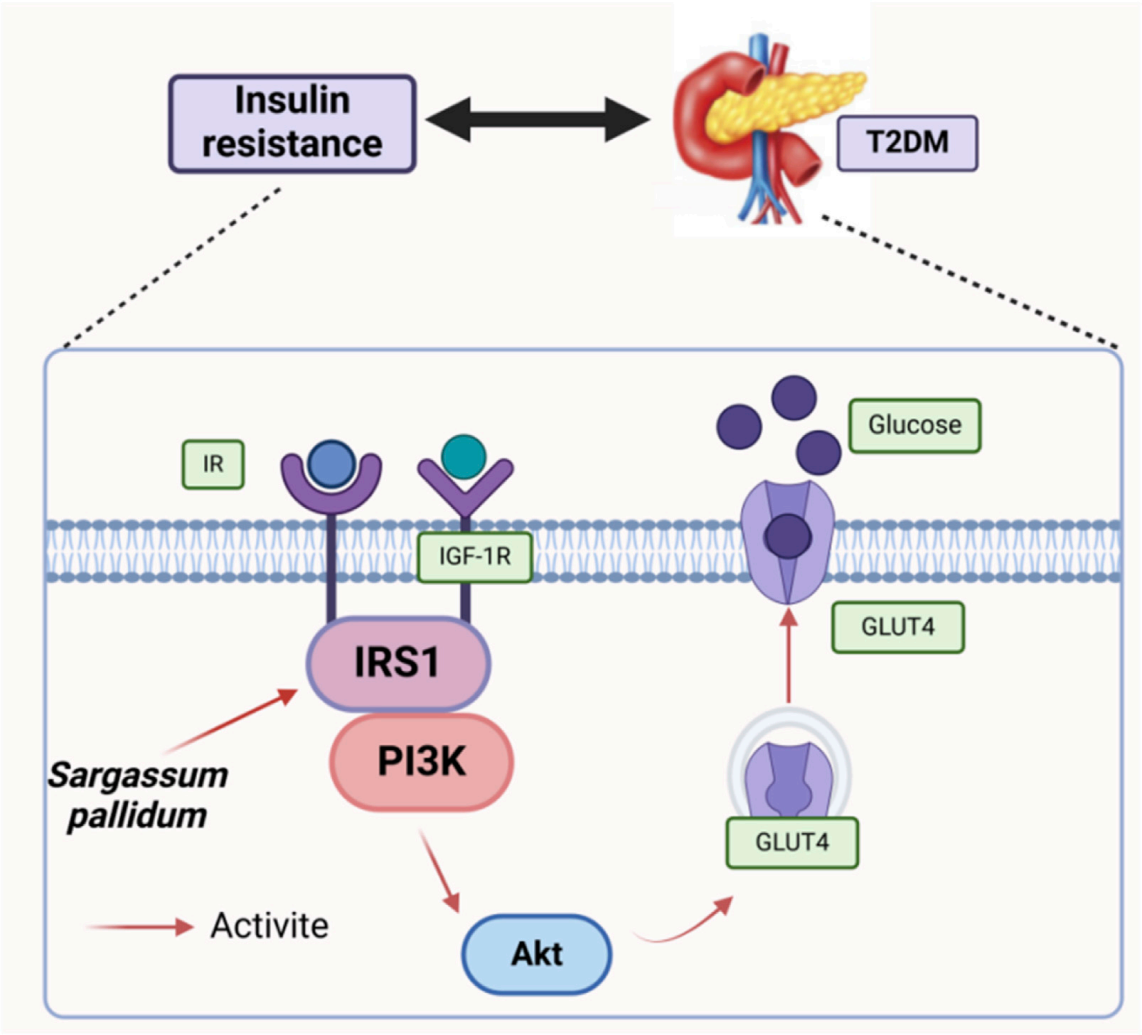
Main mechanistic pathway of blood glucose regulation^2^.

Insulin resistance (IR) is a common characteristic of type 2 diabetes mellitus, in which peripheral tissues show a diminished response to glucose metabolism, resulting in abnormal glucose homeostasis (Nolan Christopher et al., 2015). The IR-HepG2 cell model was used to evaluate the hypoglycemic activity of polysaccharides, which found that SPP, along with its derivatives SPP 1-4, SP-SPP 1-6, and S-SPP 1-8, promoted the glucose consumption in IR-HepG2 cells at 2 mg/mL (Xiao et al., 2019b). Notably, glucose consumption in SPP-1 and SPP-2-treated HepG2 cells increased significantly with higher concentrations. Among them, the glucose consumption of the SPP-1-treated group was greater than that of the metformin-treated group, suggesting that SPP-1 can effectively improve glucose metabolism in HepG2 cells (Cao et al., 2018). Taking all this together, SP regulates the IRS1/PI3K/Akt pathway to maintain glucose homeostasis on the one hand (Figure 8) and the intestinal flora such as *Lactobacillus* and *Clostridium* to improve blood glucose and lipid levels on the other. However, the potential link between these two regulatory processes has not yet been clarified, representing a promising direction for future attention.

### 4.5 Antioxidant effects

Studies have shown that polysaccharides generally enhance the activity of antioxidant enzymes in the body (Zhang R. et al., 2020). Among various algal crude polysaccharides, SPPs demonstrated the most potent ability to scavenge DPPH and superoxide radicals (Cao et al., 2019b; Wang X. et al., 2020). Not only that, the scavenging of the above free radicals by sulfated S-SPP1-4, S-SPP1-6, and S-SPP1-8 was found to be dose-dependent (Xiao et al., 2019a). SPPs exhibited scavenging effects on the lipoprotein of yolk (LPO), hydroxyl radical (·OH), and superoxide anion radical (O_2_^−^·), with the scavenging rate increasing in correlation to the mass concentration of the polysaccharides (Fang and Tang, 2011). Fernando exposed human keratinocytes to UVB and found that fucoidan inhibited UVB-stimulated oxidative stress in these cells, exerting cytoprotective effects. The protective mechanism may involve the nuclear transcription factor-κB (NF-κB) and mitogen-activated protein kinase (MAPK) pathways. In addition, fucoidan can activate the production of nuclear factor E2-related factor 2 (Nrf2)-mediated antioxidant enzymes, thereby exerting antioxidant effects (Fernando et al., 2020). Based on this study, Jayasinghe demonstrated the antioxidant effects of fucoidan from SP against TNF-α/IFN-γ stimulation. The experimental results showed that SPPs significantly reduced reactive oxygen species (ROS) production induced by TNF-α/IFNγ stimulation in HaCaT keratinocytes dose-dependently at 15.6, 31.3, and 62.5 μg/mL concentrations. The treatment also suppressed phosphorylation of cytosolic IκBα, NF-κB p65, and nuclear translocation of NF-κB p65, which was mediated by activation of the Nrf2/HO-1 signaling pathway. N-acetylcysteine (NAC) was used as a positive control for ROS scavenging, but it was found to be less effective than the former (Jayasinghe et al., 2022). Lin et al. similarly suggested that SP fucoidan significantly increased antioxidant function, possibly through Nrf2/ARE and NF-κB signaling (Lin et al., 2023). Taken together, these findings indicate that Nrf2 and NF-κB signaling pathways are the primary mediators of the antioxidant effects exerted by SP. *In vivo* experiments showed that the aqueous extract of SP and SPPs significantly reduced malondialdehyde (MDA) levels while enhancing levels of superoxide dismutase (SOD) and glutathione peroxidase (GSH-Px) (Liu and Meng, 2004; Zhang et al., 2012). Meanwhile, the aqueous extract of SP increased the levels of catalase (CAT) and glutathione (GSH) in a dose-dependent manner, while maintaining normal antioxidant enzyme activities (Zhang et al., 2012). Dong et al. proposed that seaweed Yuhu Tang, which combines SP with liquorice, could also exhibit these antioxidant effects by activating the Nrf2 signaling pathway to alleviate oxidative stress in the liver. This combination was found to be more effective than each ingredient used separately, suggesting that SP and liquorice, when used in combination, could help alleviate liver diseases and provide a valuable reference for future research in this area (Dong et al., 2024).

**FIGURE 9:**
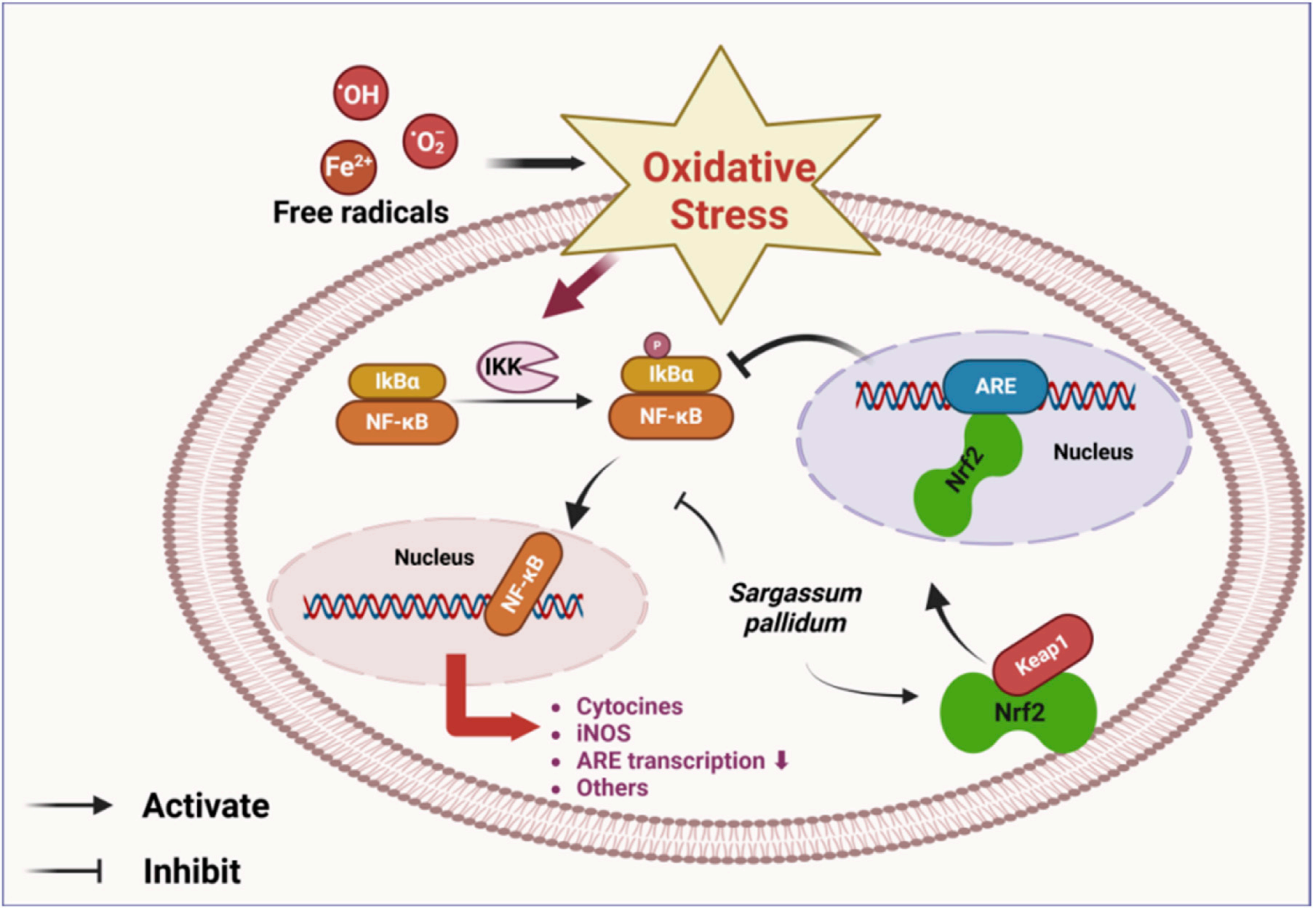
Main mechanistic pathway of antioxidation^2^.

Studies on the activity of SP polyphenols have focused on their antioxidant properties (Ye et al., 2009; Aminina et al., 2020). Results show that the free radical scavenging ability of SP polyphenols in the HepG2 cell model was significantly greater than that of other tested compounds. This suggests that polyphenols are the most crucial antioxidants in SP (Xie et al., 2023; Xie et al., 2024), providing a scientific basis for its use as a natural antioxidant. SP demonstrated potent antioxidant activities both *in vivo* and *in vitro*, with the Nrf2 and NF-κB signaling pathways identified as the primary mediators of these effects (Figure 9). Nevertheless, the detailed mechanisms underlying its antioxidant activity and the associated upstream and downstream effects require further investigation.

### 4.6 Immunomodulatory effects

It is generally believed that plant polysaccharides play a significant role in regulating the body’s immune function, enhancing both cellular and humoral immune functions. Simultaneously, they promote the growth of the body’s immune organs, activate immune cells, and release cytokines, to maintain the body’s immune balance and the dynamic balance of its physiology (Zhang Q. et al., 2020). SPP could promote nitric oxide (NO) production in macrophages within the 10–500 μg/mL concentration range. It also enhanced the proliferation of mouse splenic lymphocytes and synergized the lymphocyte proliferative response induced by ConAor LPS (Li, 2015). The SP aqueous extract had protective effects against nitrosoguanidine (MNNG)-induced immune damage in rats. After continuous administration (400, 600, 800 mg/kg) for 8 weeks in rats, the SP aqueous extract increased levels of interleukin-2 (IL-2), interleukin-4 (IL-4), and interleukin-10 (IL-10) and significantly reduced the release of interleukin-6 (IL-6), interleukin-1β (IL-1β), and tumor necrosis factor-α (TNF-α) (Zhang et al., 2012). RAW264.7 cells are a good model to study polysaccharide immunomodulatory activity. SPP could promote the proliferation of mouse macrophages at concentrations ranging from 25 to 400 μg/mL in a concentration-dependent manner. The polysaccharide fraction SPP-0.7 exhibited the highest immune cell proliferation activity at 400 μg/mL (Gao et al., 2021). Both PCR and ELISA assays revealed a marked increase in the relative mRNA expression and release of immune-related cytokines such as IL-1β, IL-6, inducible nitric oxide synthase (iNOS), and TNF-α (Gao et al., 2021), suggesting that SPP enhances the immune function (Li et al., 2020).

In experiments conducted on chickens, the immunomodulatory effects of SPP concerning infectious bronchitis (IB), Newcastle disease (ND), and avian influenza (AI) were examined. Following vaccinations on days 7, 14, 21, and 28, the SPP group exhibited higher antibody potency against ND-HI, IB-HI, and AI-HI compared to the control group, particularly at doses of 30 and 50 mg/mL, with the highest potency at 30 mg/mL (Li et al., 2012). CD4^+^ and CD8^+^ T lymphocytes serve as crucial components in immune regulation. A high CD^4+^/CD^8+^ ratio indicates activated immune function within the normal range, whereas disturbances in this ratio can lead to various immune disorders. In the SPP group, the percentage of CD^4+^ T lymphocytes was higher throughout all time periods, while the CD^8+^ T lymphocyte percentage was lower, resulting in an increased CD^4+^/CD^8+^ ratio. These findings indicated that vaccination in the SPP group was more effective and improved cellular immunity (Li et al., 2012). Collectively, SP can function as an immunomodulator, potentially exerting therapeutic effects on a wide range of diseases. The immunomodulatory effects of SP include regulating the balance of immune response, inducing the release of immunoreactive substances, and enhancing the function of immune cells. However, studies on the mechanisms of immunomodulation, such as PI3K/Akt and MAPK/NF-κB signaling pathways, have not been carried out promptly, resulting in the study of this activity remaining in the preliminary stage.

### 4.7 Other effects

Wang et al. found that SP extracts promoted the germination rate and seedling growth of wheat. The fresh weight of the seedlings and seedling root growth dramatically increased compared with the clear water control group (Wang Y. X. et al., 2020). Yang et al. discovered that the SP extract had significant anticoagulant effects on rabbits *in vivo* and *in vitro*, enhancing their fibrinolytic activity (Yang et al., 2000).

In recent years, group studies on SP have emerged. Seaweed Jade Pot Soup, first published by Chen Shigong in the Ming Dynasty, is a representative formula for the treatment of gall tumors with gas stagnation and phlegm condensation (Chen, 2011) and is commonly used clinically for the treatment of thyroid disorders such as simple goiter and thyroiditis (Bai et al., 2019; Wang and Zhang, 2021; Tian et al., 2022). Research using a rat goiter model indicated that each treatment group positively affected pathological manifestations, such as the disorganized lobular arrangement of thyroid tissue and follicle size discrepancy. Among the groups, the formula containing SP showed the most significant improvement. In addition, each treatment group increased the expression levels of Bax mRNA in thyroid cells of the model group without affecting Bcl-2 mRNA expression. These findings suggest that SP may ameliorate goiter by promoting Bax gene expression, enhancing the binding of Bax protein to Bcl-2 protein, increasing the Bax/Bcl-2 ratio, and inhibiting apoptosis (Zhang et al., 2019b).

Xie et al. investigated the antiglycation capacity of the SP extract on ovalbumin (OVA) glycation and its active metabolites, including 6-gingerol (6G) and poricoic acid A (PA). The results showed that the SP extract, PA, and 6G had excellent suppression on the formation of fructosamine, 5-hydroxymethylfurfural (5-HMF), acrylamide, and advanced glycation end products (AGEs), which was higher than that of aminoguanidine (AG). PA exhibited the most vigorous inhibition activity for protein glycation products compared with the control group (Xie et al., 2021). These findings suggested that SP extracts in healthy food had great potential as antiglycation inhibitors to treat diabetes complications. Gut microbes are involved in the energy metabolism of the host (Ralston et al., 2017; Gao et al., 2019), and dysregulation of the composition and structure of the gut flora is one of the pathogenic mechanisms of obesity and its associated metabolic diseases (Wang et al., 2019). Previous studies have shown that polysaccharides derived from natural plant resources can achieve the regulation and amelioration of chronic metabolic diseases through various mechanisms such as modulation of chronic inflammatory responses, gut microbial components, and structure (Mayengbam et al., 2019; Ferreira et al., 2022). Yuan et al. investigated the effects of SPPs on gut flora using *in vitro* simulated digestion and human fecal fermentation models. It was found that SPP could regulate gut health by decreasing the Firmicutes/Bacteroidetes (F/B) ratio and increasing the abundance of beneficial bacteria such as *Phascolarctobacterium*, *Ruminococcus*, and *Bacteroides* (Yuan et al., 2020b). Cao et al. similarly found that it helped increase the diversity of the intestinal microbial communities, especially in the pre-fermentation period. Therefore, it suggests that SPP promotes the composition and abundance of beneficial gut flora (Cao, 2021). Yuan et al. established a high-fat diet (HFD)-induced obese mouse model. The exploration indicated that SP degradable polysaccharide (UD-SPP) dramatically downregulated the expression of liposynthesis genes in the liver of obese mice, including PPAR-γ, Srebp-1c, ACC1, and FAS (Yuan et al., 2022). At the same time, NF-κB inflammatory signaling pathways, as well as the secretion of downstream IL-1β, IL-6, and TNF-α pro-inflammatory factors in the colon of obese mice, were also inhibited in mice. Subsequent mechanistic studies revealed that UD-SPP reduced obesity and inhibited intestinal inflammatory responses, which mainly relied on its regulatory effects on the intestinal flora, such as increasing the abundance of genera involved in BA metabolism and regulating the levels of genera of some glycolipid metabolism-related amino acids (Yuan, 2022).

## 5 Conclusion and perspectives

*Sargassum pallidum* (Turn.) C.Ag.*,* a traditional marine medicine, exhibits softening, expectorant, diuretic, and antidiarrheal effects. Modern research has revealed that SP contains diverse chemical metabolites such as polysaccharides and flavonoids. Its active metabolites and extracts demonstrated remarkable biological activities, including antioxidant, hypoglycemic, antitumor, antibacterial, as well as immunomodulatory properties. However, despite the widespread use of seaweeds in various applications, more comprehensive and systematic studies are needed to further investigate the research and application of SP.

Currently, the existing pharmacological studies focus on single experimental models, which provide insufficient evidence to clarify the effectiveness of treatments. It would be more scientifically sound to evaluate their activity through a combination of *in vivo* and *ex vivo* phases alongside clinical studies. Despite its long history as a traditional Chinese medicine, there is a significant lack of data regarding the safety of SP, highlighting the urgent need for toxicological research studies. Previous research has primarily concentrated on the antioxidant, hypoglycemic, and antitumor properties of SP, with minimal attention paid to its functional mainstays. As a traditional Chinese medicine derived from marine algae, SP has been widely employed in goiter and mammary gland hyperplasia. However, there is a shortage of contemporary pharmacological explanations for its traditional applications. Consequently, subsequent research could prioritize investigating the indications linked to its conventional applications. Although the rich biological activities of SP have been documented, its clinical applications remain limited. Moving forward, based on the findings from basic research, more attention should be directed toward exploring the potential clinical applications of SP to enhance its value. For instance, the immunomodulatory effects of SP suggest its potential therapeutic application in immune-related diseases.

In terms of the functional metabolites of SP, numerous studies have primarily focused on polysaccharides and crude extracts. However, there is a significant lack of research regarding the activity of other chemical metabolites, which impedes the identification of active ingredients linked to pharmacological effects. To address this, the separation and identification of metabolites should be combined with network pharmacology, data mining, and virtual screening to predict their efficacy, as well as the related metabolic targets, receptors, and pathways. This approach can then lead to experimental validation, maximizing the therapeutic potential of SP. Furthermore, the mechanism behind its pharmacological activity remains poorly understood, and the active targets and pathways associated with various disease states remain to be fully elucidated. It has been mentioned that the antitumor activity of SP is linked to apoptosis; however, the specific pathways involved require further investigation. Similarly, SPPs exhibit significant immunomodulatory effects, yet the current research mainly centers on regulating immune factors. How immune function can be regulated by mediating the corresponding signaling pathway may be a promising direction for future studies. In addition, it has been pointed out that the Nrf2, a classic antioxidant pathway, may be involved in regulating glucose and lipid metabolism upon activation. This is closely related to the hypoglycemic and antioxidant biological activities of SP, indicating that it is a key pathway in which SP may exert its effects and should not be overlooked.

Furthermore, the structural studies of metabolites and their relationship with biological activities require further refinement. It is essential to determine which specific groups within the molecule are important for conferring biological functions. This can be achieved through comprehensive structural analyses, such as 2D NMR. The connection between the conformational characteristics of polysaccharides and their biological activities remains unclear, particularly concerning their antitumor and immune-enhancing properties. Additionally, structural modifications of metabolites can be conducted using pharmacophore modeling technology, which may serve as a reference for developing novel pharmaceutical agents. In particular, the literature has widely reported that the hypoglycemic and antioxidant activities of selenized and sulfated polysaccharide–protein complexes (SPPs) are significantly enhanced following these modifications. Therefore, it can be concluded that structural modification is crucial for the enhancement of the physicochemical properties and biological activities of polysaccharides.

Moreover, the standardization of the harvesting and processing methods of SP, along with the quality control measures, warrants further investigation. Variations in processing techniques can lead to alterations in chemical metabolites; for instance, different drying methods may result in divergent outcomes during metabolite identification compared to conventional approaches. With advancements in analytical techniques and quality control methods, new quality markers and assessment measures can be employed to assess the quality of drugs more accurately.

Although SP is known for both its medicinal and edible properties, most recent research studies have primarily concentrated on its medicinal aspects, largely ignoring its potential as an edible plant. The study pointed out that SPPs can help regulate intestinal flora and the bacterial levels of certain amino acids associated with glycolipid metabolism. This finding suggests that SPP could be developed as a food ingredient aimed at improving obesity and intestinal health, showcasing the dual benefits of SP as a traditional Chinese medicine. Therefore, future considerations should focus on exploring its development into a healthcare product alongside its existing applications.

In conclusion, despite extensive research on the chemical metabolites and biological activities of SP, it offers a solid scientific basis for its effectiveness and clinical applications. However, additional analysis regarding the structure, efficacy, safety, and quality control of SP is necessary to establish a framework for future resource development and clinical utilization.

## Footnotes

2 Created with BioRender.com
